# From river blindness to river epilepsy: Implications for onchocerciasis elimination programmes

**DOI:** 10.1371/journal.pntd.0007407

**Published:** 2019-07-18

**Authors:** Robert Colebunders, Joseph Nelson Siewe Fodjo, Adrian Hopkins, An Hotterbeekx, Thomson L. Lakwo, Akili Kalinga, Makoy Yibi Logora, Maria-Gloria Basáñez

**Affiliations:** 1 Global Health Institute, University of Antwerp, Antwerp, Belgium; 2 Neglected and Disabling Diseases of Poverty Consultant, Kent, United Kingdom; 3 Neglected Tropical Diseases Control Programme, Ministry of Health, Kampala, Uganda; 4 National institute for Medical Research, Ministry of Health, Dar es Salaam, Tanzania; 5 Neglected Tropical Diseases Unit, Ministry of Health, Juba, South Sudan; 6 London Centre for Neglected Tropical Disease Research and MRC Centre for Global Infectious Disease Analysis (MRC-GIDA), Imperial College London, London, United Kingdom; University Hospital Bonn, GERMANY

## Overview

Current onchocerciasis elimination programmes do not include identification and management of onchocerciasis-associated epilepsy (OAE) in their strategies. Creating awareness about OAE will increase community-directed treatment with ivermectin (CDTI) adherence, particularly in areas of high prevalence, while motivating funders and stakeholders not to relent their efforts in the fight against onchocerciasis. Strengthening onchocerciasis elimination efforts should be prioritised wherever epilepsy prevalence is high in order to reduce OAE-related morbidity and mortality. In such areas, alternative treatment strategies including biannual CDTI, ground larviciding of blackfly breeding sites, and/or treatment with moxidectin should be considered. Addressing the OAE disease burden in these generally remote onchocerciasis-endemic regions confronted with poverty, weak healthcare infrastructures, and insecurity goes beyond current onchocerciasis elimination plans. New strategies with appropriate budgets are required. A morbidity management and disease prevention (MMDP) strategy, fully integrated into the health system, must be developed by multidisciplinary working groups involving neglected tropical disease (NTD) and epilepsy specialists, advocacy experts, and persons from affected communities. ‘River epilepsy’ needs to be urgently recognised and placed in the international development and NTD agendas.

## Policy platform

The possibility of an association between onchocerciasis (river blindness) and epilepsy (leading to the term ‘river epilepsy’; https://en.ird.fr/the-media-centre/scientific-newssheets/onchocercosis-or-river-epilepsy) has been suggested for a long time [1], but this link has not yet been fully embraced by the scientific community and stakeholders involved in onchocerciasis control. Consequently, intervention programmes targeting elimination of onchocerciasis as a public health problem do not take into account OAE. Table 1 summarises the OAE criteria [2].

**Table 1:** Updated criteria for OAE [2].

**1.**	History of two or more unprovoked seizures, at least 24 hours apart (ILAE* definition of epilepsy)
**2.**	Onset of epilepsy between 3 and 18 years of age
**3.**	Normal psychomotor development prior to epilepsy onset
**4.**	No obvious cause of epilepsy identified in the individual during the 5 years preceding seizure onset, such as perinatal brain insult, head trauma, or previous infection of the central nervous system
**5.**	At least 3 years of residence in an onchocerciasis-endemic village with high epilepsy prevalence** and frequent household clustering of persons with epilepsy

All five criteria must be met. Additional arguments in favour of OAE include a positive skin snip for *Onchocerca volvulus* microfilariae or seropositivity to Ov16 antigen confirming exposure to *O*. *volvulus*, head-nodding seizures and/or severe unexplained stunting with delayed or absence of external signs of sexual development, or presence of persons with such features in the village.

* ILAE: https://www.ilae.org/.

** An epilepsy prevalence threshold is not included because determining whether the point prevalence of epilepsy is above such a threshold would require conducting an epilepsy prevalence study, which can be complicated and costly or beyond what is feasible and may therefore lead to underreporting of OAE. However, if the epilepsy prevalence is known, a prevalence >2% should be considered as a high prevalence, given that the median epilepsy prevalence in sub-Saharan Africa is 1.4% [24].

**Abbreviations:** ILAE, International League Against Epilepsy; OAE, onchocerciasis-associated epilepsy.

In this paper, we make the case that incorporating epilepsy diagnosis and management in patient care strategies is vital for the public health burden of onchocerciasis to be truly eliminated. Furthermore, we argue that most such activities can be integrated into primary healthcare using community drug distributors (CDDs), the cornerstone of onchocerciasis elimination strategies.

## Evidence that onchocerciasis causes epilepsy

There is increasing epidemiological evidence that onchocerciasis is, directly or indirectly, a cause of epilepsy [2]. The strongest evidence to date was recently obtained in a retrospective cohort study conducted in the Mbam valley in Cameroon, which showed that the risk of developing epilepsy later in life was positively associated with skin microfilarial density in early childhood, demonstrating not only a temporal directionality from *Onchocerca volvulus* infection to subsequent epilepsy but also a strong dose–response relationship [3].

Controlling onchocerciasis has been observed to alter the epidemiology of OAE in affected communities [2]. In 2013 in Uganda, no new cases of nodding syndrome (NS, one of the presentations in the clinical spectrum of OAE) were reported one year after implementation of (biannual) CDTI, coupled with ground-based larviciding of the blackfly vector riverine breeding sites [4]. In contrast, in South Sudan, where CDTI has frequently been interrupted and no complementary interventions have ever been implemented, there is an ongoing NS epidemic [5].

Despite convincing epidemiological evidence suggesting that *O*. *volvulus* infection causes epilepsy, the pathophysiological mechanisms remain to be determined [6]. This, however, does not justify inaction while communities and individuals remain seriously affected by this condition. Although experimental and epidemiological research must continue, the existing evidence compels urgent action to prevent children from developing OAE in onchocerciasis foci where *O*. *volvulus* transmission persists despite decades of CDTI or where CDTI is yet to be effectively implemented [2].

## Disease burden caused by OAE

Regardless of opinions on causality, OAE is an important neglected public health problem, placing a large burden on patients and their caretakers. It has been estimated that in 2015, c. 381,000 people could be affected by OAE across all onchocerciasis-endemic areas [7]. Moreover, OAE may increase onchocerciasis-related mortality among children and adolescents. In a 25-year follow-up study of 295,909 individuals in the Onchocerciasis Control Programme (OCP) in West Africa, not only did the relative risk of mortality increase with skin microfilarial density but also, for a given density, this risk was statistically significantly higher in individuals younger than 20 years than in those aged above 20 [8]. Although this needs further investigation, this excess mortality under 20 years could be due, at least in part, to the consequences of epileptic seizures associated with onchocerciasis; indeed, children usually develop OAE at a peak age of 8–11 years [2], while river blindness generally occurs after the age of 20 [9]. Onchodermatitis may develop earlier, but it is not a fatal condition.

In the Mahenge area in Tanzania, where precontrol onchocerciasis prevalence by nodule palpation of adult males was 79%, a recent study revealed a 3.5% epilepsy prevalence (with the majority of persons with epilepsy [PWE] meeting the OAE criteria in Table 1) in two rural villages despite 20 years of CDTI [10]. In these villages, Ov16 seroprevalence among the 6–10 year old was 43%, indicating ongoing transmission [10]. This was confirmed by entomological findings showing *O*. *volvulus* infection rates in *Simulium damnosum* s.l. blackflies similar to those reported in the 1960s [11]. In South Sudan, a recent epilepsy survey in Maridi county showed an epilepsy prevalence ranging from 3.5% to 6% in villages close to the Maridi river and 12% in a village located close to the Maridi Dam [12], a blackfly breeding site, with 85% of PWE meeting the OAE criteria [13].

## Current onchocerciasis elimination programmes

Major progress has been made concerning the control of onchocerciasis, initially by the OCP (1974–2002) and subsequently by the African Programme for Onchocerciasis Control (APOC, 1995–2015) and the Onchocerciasis Elimination Program for the Americas (OEPA, 1993–ongoing) [14]. New cases of blindness and severe skin disease are rare except in conflict zones, where instability has had a detrimental impact on programmes’ performance, and in areas of difficult access [15]. CDTI has indeed been very successful in many areas, and since 2010, the paradigm of onchocerciasis control has shifted to that of elimination [16].

While no country in Africa has yet been verified free of the disease, in some areas of countries including Ethiopia, Mali, Niger, Nigeria, Senegal, Sudan, and Uganda, CDTI has stopped or is close to stopping [14]. National onchocerciasis elimination expert committees (NOECs) are being established in all onchocerciasis-endemic countries [17]. The World Health Organization (WHO) has proposed 2025 as a target for onchocerciasis elimination in 80% of African countries [16]. However, many unresolved challenges remain before reaching this goal [17]. Although CDTI has significantly reduced onchocerciasis-associated morbidity in many regions, there is still active transmission in many others despite long-term CDTI [10,18].

Present onchocerciasis elimination programmes focus on preventive chemotherapy with ivermectin to decrease transmission. In contrast, the Global Programme to Eliminate Lymphatic Filariasis (GPELF) has adopted a two-pronged approach that, in addition to deploying mass drug administration (MDA), has developed a strategy for MMDP [19]. For lymphatic filariasis (LF), a basic package of care, rehabilitation, and psychosocial support integrated into primary healthcare has been devised, and targets for its implementation have been proposed [19]. Despite the high disease burden caused by OAE, related morbidities and disabilities remain unaddressed.

## Implications for onchocerciasis elimination programmes

Current onchocerciasis elimination strategies need to be revised and retargeted, taking OAE into account. As onchocerciasis-related blindness is gradually disappearing, it has been more difficult to maintain interest and financial support for CDTI programmes. If the 2025 elimination goals are to be met, ivermectin coverage must be increased, and people should be encouraged to continue taking the drug. Creating awareness and community engagement about OAE is paramount for increasing adherence to CDTI and for policy makers in the health sector to strengthen their onchocerciasis elimination strategies [4,20].

ESPEN, the WHO AFRO Expanded Special Project for the Elimination of NTDs, which has taken over some of the functions of APOC, is currently working on mapping all areas not yet under ivermectin treatment that may need to be included in the transition from control to elimination. During such mapping efforts, the prevalence of OAE could be assessed by using a simple OAE clinical case definition as proposed in Table 1. If the proportion of PWE meeting the OAE criteria in a population is substantial, this is highly indicative that onchocerciasis is a major cause of epilepsy in this community. The main limitation, in the absence of complementary tests, resides in being able to differentiate OAE from neurocysticercosis (NCC). However, the peak age of epilepsy onset in persons with NCC is generally above 20 years [9], while for OAE, this is between the ages of 8–11 years [2]; also, NCC more often presents with a focal neurological deficit [9].

Strengthening onchocerciasis elimination efforts should be prioritised wherever epilepsy prevalence is high (>2%, Table 1) and transmission (by assessment of prevalence of anti-Ov16 antibodies in children and/or of infection/infectivity in blackfly vectors [20]) is ongoing. In such areas, ivermectin coverage surveys should be conducted. If coverage/adherence is insufficient (at least 80% of the eligible population is required [21]), innovative strategies to improve and sustain it should be developed, and alternative treatment strategies (ATSs), including biannual CDTI, local vector control, and/or MDA with more potent microfilaricides (e.g., moxidectin), should be considered [16]. If coverage/adherence is sufficiently high, the possibility of suboptimal ivermectin responses, as documented in Ghana and Cameroon [22], should be investigated.

Clinical trials should be conducted to determine whether it is safe to administer ivermectin (or moxidectin) in the under-fives. Although children generally develop OAE above the age of 5 years, OAE onset has been reported even around the age of 3 [2]. Additionally, increasing MDA frequency would help curtail transmission and better protect this age group.

## Epilepsy management in onchocerciasis-endemic regions

Although some countries have strategies for controlling epilepsy, these are rarely implemented outside of large cities and certainly not in the more remote onchocerciasis-endemic areas. Local epilepsy management must, therefore, depend on local nonphysician staff such as nurses working at primary healthcare facilities or community workers; this strategy is currently being encouraged by the Mental Health Gap Action Programme (mhGAP) of WHO (https://apps.who.int/iris/bitstream/handle/10665/250239/9789241549790-eng.pdf;jsessionid=6010FA3D98E95B3003B61D3C30184F0C?sequence=1), targeted to nonspecialized healthcare providers working at first- and second-level healthcare facilities. These personnel would need to be trained in case identification and simple management protocols. Follow-up could easily be undertaken by CDDs. These CDDs can also be engaged for sensitisation at the community level to enhance antiepileptic treatment adherence by PWE, and their treatment reports would facilitate regular reordering of the necessary supplies. These extra duties could be included in CDD retraining sessions at relatively little extra cost.

## Examples of how OAE research-based advocacy can drive policy to strengthen onchocerciasis elimination programmes

1. Direct representation to the President of Uganda regarding NS resulted in implementation of both biannual ivermectin treatment and larviciding of vector breeding grounds. As a result, NS incidence in Uganda has been brought down to zero [4].
2. Based on the findings in Mahenge [10] and subsequent recommendations from the Tanzania NOEC, the national NTD control programme decided to introduce biannual ivermectin distribution in the area and is considering ground-based larviciding of rivers in Mahenge and other foci with persistent transmission.
3. Based on the study in Maridi county, South Sudan [12], the Ministry of Health is planning to survey blackfly breeding sites and to implement biannual CDTI in the region. Training of CDDs involved in the next MDA round to recognise and report epilepsy as a disability associated with onchocerciasis is also being considered.

**Fig 1:**
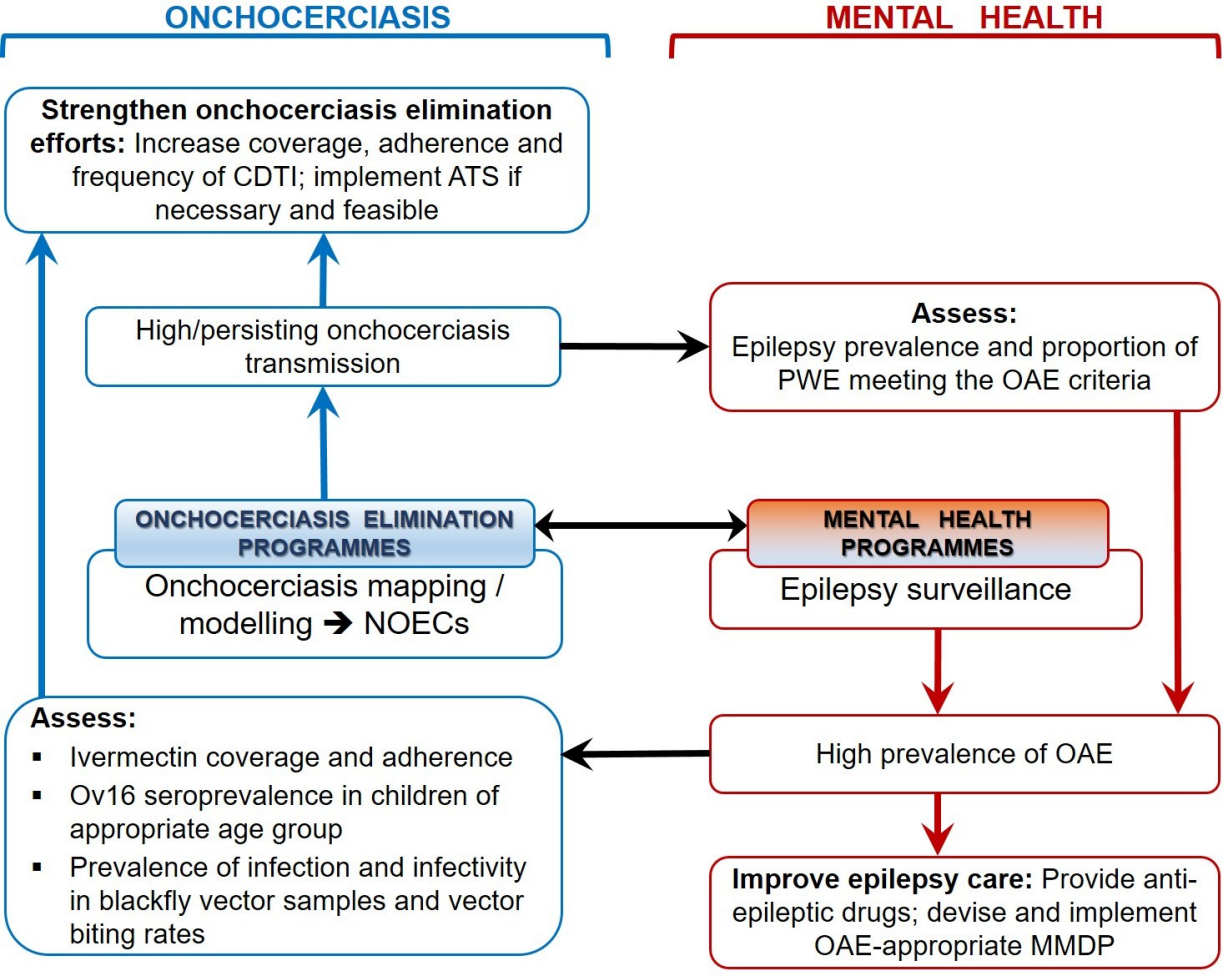
Collaboration between onchocerciasis elimination and mental health surveillance programmes. In the proposed framework, onchocerciasis mapping efforts should encompass OAE surveillance using simple criteria (Table 2). High onchocerciasis prevalence and/or ongoing transmission warrants reinforcement of onchocerciasis elimination and epilepsy assessment. Detected epilepsy hotspots should become targets for improved management strategies in collaboration with mental health experts. ATS, alternative treatment strategy; CDTI, community-directed treatment with ivermectin; MMDP, morbidity management and disability prevention; NOEC, national onchocerciasis elimination committees; OAE, onchocerciasis-associated epilepsy.

These examples illustrate the importance of i) investigating and documenting epilepsy in onchocerciasis meso- and hyperendemic foci, ii) channelling these findings through NOECs to the Ministries of Health and appropriate authorities, iii) establishing collaborations between NTD and mental health programmes, and iv) leveraging additional resources necessary to treat PWE and strengthen existing onchocerciasis elimination programmes (Fig 1).

**Table 2:** Challenges, opportunities, and needs to fully recognise the public health importance of OAE and use its advocacy to strengthen onchocerciasis elimination programmes.

Challenge	Needs
(1) Strengthen the evidence base to establish causality between *O*. *volvulus* infection and epilepsy	1) Conduct fundamental research to establish causality and pathophysiological mechanism(s) of seizures, including NS [6]
(2) Put OAE on the international development aid agenda	2) Involve affected persons/communities, local leaders, and researchers in advocacy. In contrast with the notion that onchocerciasis no longer represents a public health concern, its substantial burden of disease due to OAE morbidity and mortality should be quantified and recognised
(3) Leverage funds and human resources, including community mobilisation, to implement biannual CDTI and vector control activities (including insecticidal and noninsecticidal approaches)	3.1) Conduct prospective studies to investigate whether increasing CDTI frequency, with or without vector control (including noninsecticidal, community-directed antivectorial measures [25]), will decrease OAE incidence and be cost-effective 3.2) Prioritise funds to decrease *O*. *volvulus* transmission in areas of high epilepsy incidence and prevalence
(4) Improve healthcare infrastructure, implement epilepsy surveillance, and train epilepsy healthcare workers	4.1) Strengthen peripheral health systems by developing community-based epilepsy treatment/care programmes 4.2) Establish collaborations and synergies between NTD and mental health programmes

**Abbreviations:** CDTI, community-directed treatment with ivermectin; NS, nodding syndrome; NTD, neglected tropical disease; OAE, onchocerciasis-associated epilepsy.

## Additional benefits of OAE advocacy: Stigma reduction

In onchocerciasis-endemic foci, it is common to find households in which several children present with epilepsy, especially in families residing and/or farming in land close to blackfly breeding sites. Intense exposure to *O*. *volvulus*-infected vectors likely puts the children in those households at an increased risk of developing OAE [2,3]. The household clustering of PWE has led communities and local healthcare workers to wrongly believe that epilepsy is contagious and transmissible by direct contact, hence increasing stigma. Therefore, educating communities and health professionals about OAE will reduce stigma and motivate people to take ivermectin [23].

## Challenges

Table 2 summarises some challenges and opportunities regarding the recognition of OAE as a public health problem.

## Conclusions

It has been stated that ‘in Africa, onchocerciasis has been eliminated as a public health problem across the length and breadth of the continent’ [14]. This may be true of river blindness, but it is certainly not for ‘river epilepsy’. In order to eliminate onchocerciasis and associated morbidity and mortality, OAE must be tackled. Existing onchocerciasis elimination frameworks cannot address the OAE burden because this requires a multidisciplinary approach and collaboration between NTD programmes, mental health programmes, local communities and funding bodies, and integration into the health system. Increasing OAE awareness, strengthening onchocerciasis elimination strategies, and developing MMDP plans are pivotal in curbing OAE. Ultimately, OAE needs to be put on the international development and NTD agendas as soon as possible. ‘River epilepsy’ must be recognised and eliminated.
